# Providing Incentives on a Smartphone-Based Mood Relapse Warning Application among Patients with Bipolar Disorder

**DOI:** 10.1192/j.eurpsy.2023.626

**Published:** 2023-07-19

**Authors:** Y.-H. Lin, F.-H. Cheng, E. C.-L. Lin

**Affiliations:** Nursing, National Cheng Kung University, Tainan, Taiwan, Province of China

## Abstract

**Introduction:**

Most of the research explored the attrition rate and predictive factors for the smartphone application of emotion monitoring in bipolar disorder patients. However, there is less focus on the efficacy of maintaining the retention rate if the incentive system is employed.

**Objectives:**

The aim of our research is to evaluate the efficacy of two different kinds of incentive systems on improving frequency of using the Smartphone Mood Relapse Warning application (MRW-APP) (Su et al., 2021) in bipolar patients.

**Methods:**

A one-group pretest-posttest pilot study was conducted. Participants with bipolar disorder (n = 63) recorded their moods and symptoms through MRW-APP for 29 weeks with the attrition rate of 44%. Two different kinds of incentive systems, reward and lottery, were implemented. To know whether incentive implementation could play a role in motivating the participants to better adhere to the app, we used Friedman’s test and paired sample t-test to analyze the participants’ app-using frequency in the corresponding weeks.

**Results:**

There was no significant difference in the participants’ app-using frequency (*p*>.05) before and after we implemented the first incentive system, reward (n=63). For the second incentive system, lottery (n=41), a significant difference in app-using frequency was still not observed (*p*>.05) after the intervention. But, for those who both had experienced two kinds of incentive systems (n=35), there were significant changes in their app-using frequency (*p*<.05).

**Table 1.** Demographics (n=63)

Variables	All (n=63)	
	Mean	*SD*
**Age (n=55)**	36.40 25.27	11.10
**Onset age (n=48)**		9.35
	n	%
**Gender (n=54)**		
Female	33	61.1
**Educational level (n=54)**		
Above undergraduates	40	74.1

SD= Standard deviation

**Image:**

**Figure:**
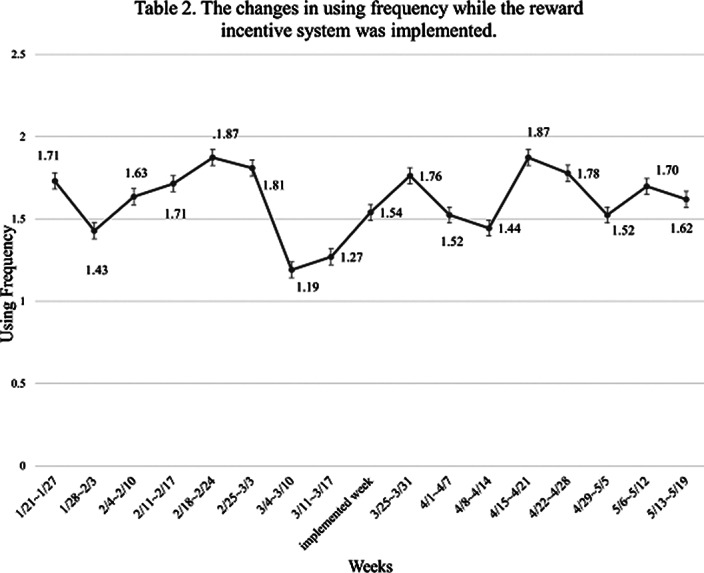

**Image 2:**

**Figure:**
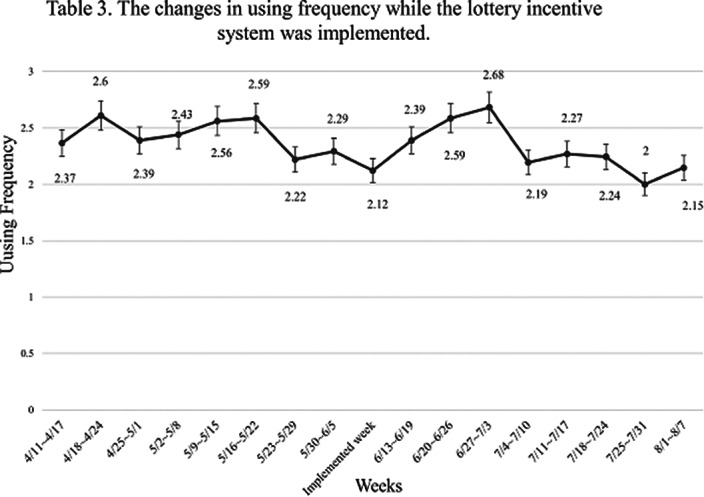

**Conclusions:**

This research found the two incentive systems, award and lottery, may help increase the using frequency of the smartphone monitoring app for participants with bipolar disorder. The results from our study can be a reference for mood monitoring apps development in the future, and it also suggested that incentive system has its potential on encouraging patients’ adherence to e-healthcare.

**Disclosure of Interest:**

None Declared

